# Control of Tungiasis in Absence of a Roadmap: Grassroots and Global Approaches

**DOI:** 10.3390/tropicalmed2030033

**Published:** 2017-07-27

**Authors:** Lynne Elson, Katherine Wright, Jennifer Swift, Herman Feldmeier

**Affiliations:** 1 Dabaso Tujengane CBO, Watamu 80202, Kenya; 2 Spatial Sciences Institute, University of Southern California, Los Angeles, CA90089, USA; kawright@gmail.com (K.W.); jswift@dornsife.usc.edu (J.S.); 3 Institute of Microbiology and Hygiene, Campus Benjamin Franklin, Charité University Medicine, 12203 Berlin, Germany; hermann.feldmeier@charite.de

**Keywords:** tungiasis, *Tunga penetrans*, jiggers, neglected tropical disease, dimethicone, neem, web GIS, elibrary, Esri, geographic information science

## Abstract

Tungiasis is a tropical skin disease caused by the sand flea *Tunga penetrans*. It inflicts misery upon tens of millions of people, mostly children, across Central and South America and sub-Saharan Africa, and yet there is no globally accepted roadmap for its control. Here we review how research in the last 15 years has developed control methods and report on new grassroots and digital mapping approaches. Treatment is now possible with a two-component dimethicone, used for the treatment of headlice in Europe, Asia and Canada, but not yet available in most tungiasis-endemic areas. Prevention is possible through the daily use of repellents based on coconut oil. A Kenyan coastal community has successfully controlled tungiasis using a neem and coconut oil mix produced locally to treat cases, combined with spraying floors with neem solution and distributing closed shoes. Development of affordable hard floor technology is underway, although not yet widely available, but is a priority to control off-host stages in the floors of homes. A new web-based digital mapping application will enable researchers and health officials to collaborate, share data and map the prevalence of tungiasis. We conclude that tungiasis can be controlled through a multi-disciplinary, One Health approach.

## 1. Introduction

Tropical parasitic skin diseases are experienced by millions of the poorest populations of the world. However, many of them are not listed by the World Health Organization as Neglected Tropical Diseases (NTDs). As a consequence they are ignored by governments and health care workers alike and they do not receive the scientific interest they merit. Tungiasis (sand flea disease) is one of them, caused by a parasitic insect, the female sand flea (*Tunga penetrans*). In spite of the lack of funding, a fair amount is known about the parasite’s ecology, epidemiology and the morbidity caused by the disease. There is no universally accepted method for treatment or prevention in endemic communities and a ‘roadmap’ for its control has never been developed.

## 2. Life Cycle

*T. penetrans* belongs to the family Siphonaptera. Only the adult female sand flea burrows into the epidermis of its host, usually on the feet, with the final segments of her abdomen protruding. The female is fertilised by a male while she is already embedded in the skin [1]. The female undergoes a remarkable hypertrophy called neosomy as the eggs develop inside her abdomen. One week after penetration, eggs will be expelled and fall on the soil or floor. Under favourable conditions, they develop in dry soil into larvae and pupae, before emerging as infective adults themselves [1]. Hence, the off-host cycle is identical to all other flea species.

## 3. Epidemiology

Around the world tungiasis has various local names including jiggers, chigoe, pique or nigua, indicating that the disease has been prevalent in many populations for a long time*.* Tungiasis has reached endemic proportions among marginalized, resource-poor urban and rural communities in sub-Saharan Africa, South America and several islands in the Caribbean [2]. Prevalence may be up to 60% in children and 30% in the general populations [3,4,5,6,7]. However, there is no data in any country of the accurate geographic distribution and age-specific prevalence. In most endemic areas transmission is seasonal, with the highest incidence during the dry season [8]. When transmission occurs indoors, incident cases appear the whole year round. 

Children, disabled persons and the elderly bear the highest disease burden [6,7]. While most cases have few embedded fleas, some individuals may present with hundreds [9,10]. A persistent high parasite load leads to complete immobilization, anaemia and cachexia [11]. Studies on the risk factors of tungiasis have consistently shown that demographic, behavioural and environmental factors associated with poverty predispose to a high disease burden [12,13,14].

Tungiasis is a zoonosis with domestic animals such as dogs, cats, goats, pigs, and sylvatic animals such as rats and monkeys being infected and serving as reservoirs, with different species being the main reservoir in different areas [1,15,16,17,18,19]. There is convincing evidence that the whole life cycle can be completed indoors without involvement of a reservoir [20].

## 4. Morbidity

The severity of the disease is directly related to the number of embedded fleas [21]. Acute morbidity is triggered by an intense inflammatory response around embedded sand fleas [22]. Bacterial superinfection is almost constant and re-enforces the inflammation [12]. In non-immunized individuals, fatal tetanus is a known complication [23,24]. Over time, with constant re-infection, chronic pathology develops characterised by desquamation of the skin, oedema around the nail rim, fissures, ulcers, deformation and loss of nails [25].

Due to pain and itching, children are unable to concentrate on their lessons at school and have difficulty in sleeping and walking [2,23,25,26]. Tungiasis has recently been demonstrated to significantly impair the quality of life of children [27]. Infection is often associated with stigma, with victims being ridiculed, and they consequently isolate themselves. Since tungiasis impairs mobility and concentration, it impacts children’s ability to learn and to get jobs [28]. Similarly, tungiasis affects adults’ ability to conduct their work, whether it be a paid job or on their own farm [29].

Tungiasis in livestock is associated with severe morbidity and by consequence reduces the merchantability of affected animals, an additional negative impact on household economics [30,31,32]. Thus, there is a vicious cycle between poverty exposing people to tungiasis and tungiasis causing more poverty and ultimately impacting the socioeconomic development of whole communities. 

## 5. Prevention

A series of studies performed in Brazil and Madagascar have convincingly shown that the twice-daily application of a repellent comprising coconut oil, aloe vera and jojoba oil to the skin of the feet effectively prevents the penetration of female sand fleas [33,34]. This was paralleled by a complete resolution of acute pathology, and partial resolution of chronic pathology, even in very severe cases [35]. Unfortunately, the repellent is no longer commercially available. However, based on its principle ingredient, coconut oil, it could be produced locally.

## 6. Treatment

Until recently, there was no effective and safe chemotherapy available. For those with access to good quality medical facilities and antiseptic follow-up care, surgical extraction of the whole flea, intact, under sterile conditions, or cryotherapy [36], has been the best treatment. 

Self-treatment by surgical extraction is also the main method used by affected individuals in endemic communities [37,38]. However, this is a health hazard, since it is conducted with thorns or other locally available sharp instruments without proper disinfection, resulting in a high risk of bacterial superinfection. Additionally, physical injury and haemorrhage caused by extraction with inappropriate instruments can expose the patient to the risk of transmission of HIV and hepatitis B and C virus when the instruments are used consecutively for multiple persons, as is common in endemic areas [38]. Additionally, rupture of the embedded flea increases the inflammatory response [39,40]. It goes without saying that surgical extraction of embedded sand fleas is extremely painful and traumatizes the child. In Kenya and Uganda, children ran away when health personnel arrived because they feared that embedded parasites would be removed surgically [20]. 

Apart from physical extraction, affected communities use other hazardous treatments including motor oil and household animal insecticides such as cattle dip [37,41]. Affected Kenyan communities use a variety of plant products crushed in carrier oil such as paraffin or coconut oil. These include cork bush (*Mundulea sericea,* mthupa), black monkey orange *(Strychnos madagascariensis,* mujaji*),* velvet leaf *(Cissampelos pareira,* kasiropaka*)*, and sodom apple (*Solanum incanum,* mtunguja mwitu*)* [42,43]. Some of these plant extracts are potentially toxic.

In Kenya, the treatment used most widely is a 15-minute soak of the feet in 0.05% potassium permanganate baths followed by drying and covering with petroleum jelly. This is not only a cumbersome and painful procedure [42], but a recent clinical trial showed that it is only marginally effective, with only 39% of embedded fleas showing signs of non-viability 7 days after treatment [44]. In addition, permanganate is toxic if consumed and therefore inappropriate for home application. 

Two randomised controlled trials have shown that embedded sand fleas can be effectively killed by the application of a synthetic oil containing two different dimethicones [44,45]. The efficacy varied between 78% and 95%. Local inflammation significantly decreased within seven days [44]. If the dimethicone is targeted to the abdominal cone of the parasite protruding above the skin, a few drops are sufficient to kill the embedded sand flea. Since the few embedded fleas that are not killed by dimethicone no longer excrete eggs, systematic application of this treatment will also have an impact on transmission. The mode of action of dimethicones is purely physical and hence, does not entail the risk of resistance developing. Dimethicone oils are completely non-toxic [46]. However, because dimethicones are inflammable, treated patients must stay away from fires for up to two hours after application (depending on the volume applied). This of course would be no problem if treatment is conducted at school. Currently, the dimethicone is only available in a few endemic areas.

## 7. Control of Off-Host Stages

Risk factor studies conducted in Brazil, Nigeria and Kenya have identified ‘living in a house with a dirt floor’ as the major risk factor for tungiasis, indicating that the off-host life cycle is frequently completed indoors. Soil surveys in Brazil found that the majority of infected samples were from inside people’s sleeping room under the bed or hammock [47]. The same was true for a pilot study performed in Kenya and Uganda [48].

Consequently, the best target for the control of off-host stages in settings where transmission occurs predominantly indoors, is replacing dirt floors by solid floors such as concrete, ceramics, bricks, and so on. However, the costs for a concrete floor are approximately US $180 for an average home in Kenya (24 m^2^), which is why the majority of families affected by tungiasis do not have them. Development of low-cost, permanent floors which could be easily cleaned every day are urgently needed. One organization leading this search is ‘Earthenable (https://earthenable.org/)’ in Rwanda, which has developed a multi-layer floor sealed with an oil. However, even this technology is out of reach of the majority of the most needy, requiring donations or a microfinance strategy to support its roll-out [49]. 

The Kenya National Policy Guidelines for Jigger Control 2014 recommend the use of insecticides, including hypercypermethrin and methylcarbamates, to chemically treat the floors. This approach is highly questionable considering the toxicity of these compounds in humans, animals and the environment. Besides, larger scale application is expensive and beyond the reach of those most in need and may rapidly lead to resistance.

## 8. Control of Animal Tungiasis

Since in most endemic areas animals act as reservoirs and animal tungiasis reduces the merchantability of livestock, a One Health approach is needed if elimination of tungiasis and reduction of poverty are set as goals. Treatment of animal tungiasis has been shown to be possible with a combination of chlorfenvinphos, dichlorphos and gentian violet [32] or a combination of imidacloprid and permethrin [50]. However, taking into consideration the weakness of veterinary health services in the endemic areas, this approach is currently not feasible, and the cost of the products puts them out of reach of affected communities.

## 9. Digital Mapping Technologies

Recent research in the public health sector acknowledges the power of open data and mapping in combatting several poverty-related diseases and conditions [51,52,53,54,55,56,57,58]. Because mapping technology has advanced so rapidly in the last decade, many epidemiologic maps with varying levels of interaction have come and gone with little lasting effect, but each new iteration builds on the successes of previous applications [56]. There is a clear need for mapping in the control of NTDs such as tungiasis; of most interest to scientists and policy-makers are maps of prevalence and maps of spatial distribution. The distinction between the two is one of semantics, yet critically important. Disease distribution maps indicate areas where a parasite or disease is known to exist, while prevalence maps indicate the frequency of disease within a population. Both are of equal importance to health care providers and scientists, but locating data for either is problematic in endemic countries.

Static maps of these variables are obsolete almost as soon as they are printed, so a dynamic web map of prevalence is of great use to researchers [59], especially one storing georeferenced prevalence studies published in scientific journals and ‘grey’ literature such as conference proceedings, state organizational publications, or old and unpublished data [60]. Academic research can be augmented with mapping technologies and techniques such as geographic information systems (GIS) and volunteered geographic information (VGI) to produce web-based applications purpose-built to support epidemiological disease management. Web mapping, also commonly referred to as web GIS, can be loosely defined as GIS or a GIS application that runs within a browser window [61,62]. The use of the internet browser to house the application means that the user experience remains the same for all users, regardless of mobile device type. 

A number of web mapping applications have been developed to store and distribute NTD data such as geographical distribution and prevalence. A primary function for these databases is to produce maps of distribution, prevalence, endemicity, and risk. The NTD Mapping Tool, (NTD MT), the Global Neglected Tropical Disease Database (GNTD), the Global Atlas of Helminth Infections (GAHI), and the Global Atlas of Trachoma (GAT) were reviewed with regard to the development of a powerful web application for tungiasis (www.ntdmap.org/; www.gntd.org; www.thiswormyworld.org; www.trachomaatlas.org). These examples support the development of publicly-available and easy-to-use web and mobile mapping applications focused on tungiasis to assist aid workers, researchers, and governments working toward its management. 

The Tungiasis Elimination Project (TEP) is a web GIS recently developed to display many different types of data representing environmental and social risk factors for tungiasis. Because of its tropical climate, largely rural, poor population, increasing construction of roads and mobility of the population, and reliance on subsistence agriculture, Kenya was chosen as an analogue for other tungiasis-endemic countries in the development of the web mapping applications. In addition, compared to other countries in sub-Saharan Africa, Kenya boasts excellent data availability through its open data portal (http://www.opendata.go.ke/). Other valuable resources include the International Livestock Research Institute (www.ilri.org), the Kenya National Bureau of Statistics (www.knbs.or.ke), the World Resources Institute (http://www.wri.org), and Virtual Kenya (www.virtualkenya.org/). Data procured from these sources represent critical risk factors associated with tungiasis; these risk factors are geographic in nature and uniquely fit for display in a GIS [12]. Using Kenya for a case study enables the web mapping concept to be scaled to other areas that share similar geographic and sociological attributes.

In future, other overlays can be added to map risk factors such as land cover, land use, temperature, humidity, rainfall and soil moisture, which play integral roles in *T. penetrans* development. Social risk factors such as poverty, literacy, alcoholism, education, sustainable access to water, sanitation and hygiene practices, access to medical treatment, housing conditions, population density, and proximity to livestock can be displayed visually within a GIS, typically aggregated at the county or district level. Arranging these data layers over each other on a map can reveal spatial patterns that are obscured when viewed as tabular data. Spatial analysis is a data-driven way to identify areas of high risk to be targeted for treatment. In addition, recording the distribution of medical supplies and implementation of treatment methods in a spatial database will permit statistical and spatial analyses across regional as well as local geographies, revealing successes and failures in treatment approaches. 

**Figure 1:**
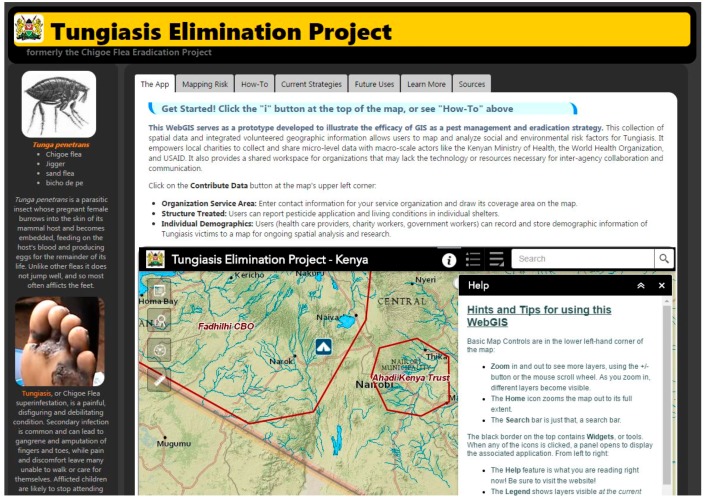
Screenshot of the Tungiasis Elimination Project Application Website.

Prior to Wright’s study (2017), no VGI-based database developed specifically for WHO tungiasis reclassification existed, nor had a web GIS been proposed to house both georeferenced socioeconomic and epidemiologic data collected by aid workers and peer-reviewed publications. Thanks in large part to recent advances in web mapping technologies, it is now possible to provide standardized GIS workflows to multiple organizations battling the same disease across great distances. The Tungiasis Elimination Project (TEP) application, shown in Figure 1, serves aid workers and non-governmental organizations (NGOs) in developing strategies to manage tungiasis [63]. Future versions of the TEP will enable field data collection using readily available mobile mapping tools such as Esri’s Collector for ArcGIS application, which can be augmented with a Bluetooth-connected global navigation satellite system receiver in areas where cellular service is not available.

The TEP application provides a collective workspace in the form of a browser-based web GIS. It offers users an opportunity to overlay 22 supporting data layers for spatial analysis, and enables the collection of georeferenced demographic and treatment data at the local level. As aid workers visit an area, they can use the TEP’s online tools to record patient demographic data and document the type of intervention used. NGOs and/or community-based organizations (CBOs) involved in tungiasis prevention and education can draw their service areas on the map and provide contact information in order to facilitate cooperation between organizations and to reveal areas in greatest need of attention. 

The Tungiasis Elimination Project and Tungiasis eLibrary web mapping applications are currently deployed through web pages hosted by the University of Southern California (Table 1).

The applications were built using GIS technologies, including Esri ArcGIS Server, ArcMap, and ArcGIS Online (AGOL). Microsoft SQL Server databases were designed specifically to house tungiasis prevalence and research data to support the applications. The web pages in which the maps are embedded are coded in HTML5, JavaScript, and cascading style sheets (CSS) to produce multi-tabbed web pages also including textual content, embedded links, and videos. 

**Figure 2:**
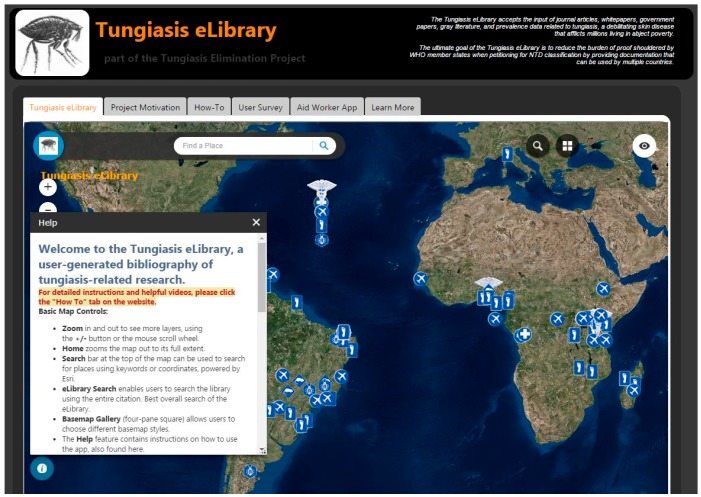
Screenshot of the Tungiasis eLibrary Application website.

The eLibrary project application (Figure 2) displays an exhaustive collection of tungiasis-related journal articles on a web map [63]. The main component of the application is a georeferenced collection of published, peer-reviewed journal articles that pertain to tungiasis or *T. penetrans*. Articles are categorized by ‘focus’ (epidemiology, entomology, public health, veterinary, travel medicine, spatial data or general information). Researchers and authors submit their own or other authors’ published work to the database as VGI by linking to other sites or uploading original documents. Users are also invited to share unpublished work, conference proceedings, government and NGO whitepapers, and even spatial data itself to further research of tungiasis eradication.

The Tungiasis eLibrary is intended to be used at a global scale and opens to an imagery basemap of the world. The dark theme is consistent with the TEP, and the logo icon is an image of *T. penetrans*. It is entitled ‘Tungiasis eLibrary’ and has a byline of ‘A user-generated bibliography of tungiasis-related literature and data.’ There are no administrative boundaries, suggesting this is a planet-wide problem. Viewing the data at this scale enables the user to see the distribution of articles, also suggesting the spatial distribution of the parasite and the disease.

The eLibrary, by mapping locations of literature pertaining to tungiasis endemicity, occurrence, and research, generates a reliable, up-to-date spatial distribution of the condition that public health professionals sorely need. Because the application can accept attachments of any kind, there is no limit to the type of data that can be collected.

## 10. A New Grassroots Approach

Endemic communities are usually able to correctly diagnose their own tungiasis [37,64] and are well aware of the impact on their children and themselves. In their desperation, a community group in the coastal area of Kilifi County in Kenya has developed its own approach to tungiasis control, using a combination of treatment and prevention in a population of approximately 30,000. The group comprises 30 community health volunteers (CHVs) who are assigned by the Ministry of Health (http://guidelines.health.go.ke:8000/media/Strategy_For_Community_Health_2014-2019.pdf) to conduct house visits in their neighbourhood to promote healthy practices and use of the health services.

**Figure 3:**
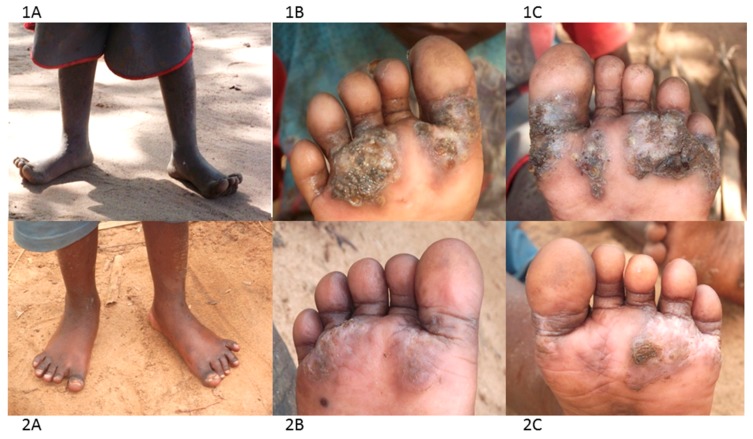
Photographs illustrating the impact of neem and coconut herbal medicine ontungiasis infection and pathology (**1A**–**C** before treatment; **2A**–**C** after 5 applications).

The group have been using a combination of treatment and prevention approaches. For treatment they use a locally-produced herbal medicine based on neem and coconut oils to treat cases, as it was available, affordable, easy to use, apparently effective and safe. CHVs regularly visit homes, local public and private nursery and primary schools, identify infected individuals and treat them. A few drops of the oil are placed directly on the embedded fleas and repeated 2–3 times in the course of one week. With time the dead flea carcass is shed by natural skin replacement processes and the chronic pathology eventually heals (Figure 3, panel 2A–C). 

The CHVs counsel children and their parents on the appropriate ways to prevent re-infection, and the dangers of extracting the fleas themselves, or using veterinary and other chemicals. Prevention methods taught include the sealing of floors, wearing of closed shoes, corralling of animals away from family resting places and keeping them out of the house, and good waste management, hygiene and sanitation practices. Another prevention tool they promote is the spraying of house floors with an aqueous solution prepared by soaking neem leaves in water for four days. Families who have sprayed their homes with neem solution report that they no longer experience the sand fleas jumping on them when they are in the house, and they no longer get newly embedded fleas [42].

In addition, the community group has partnered with TOMS^®^ (www.toms.com) to enable them to distribute free, new, simple canvas, rubber-soled shoes to children twice a year. The group distributes shoes to all of the public and private primary and nursery schools in its area of operation. CHVs inspect the children’s feet before they receive shoes and any found with embedded fleas are treated. The foot screening has been recorded to provide a simple method for monitoring the group’s impact on tungiasis prevalence and intensity. Most distribution and screening was done in May–July, the wet season, and again in September–November, the dry season. Peak transmission is during the hot and dry season in December–March. 

**Figure 4:**
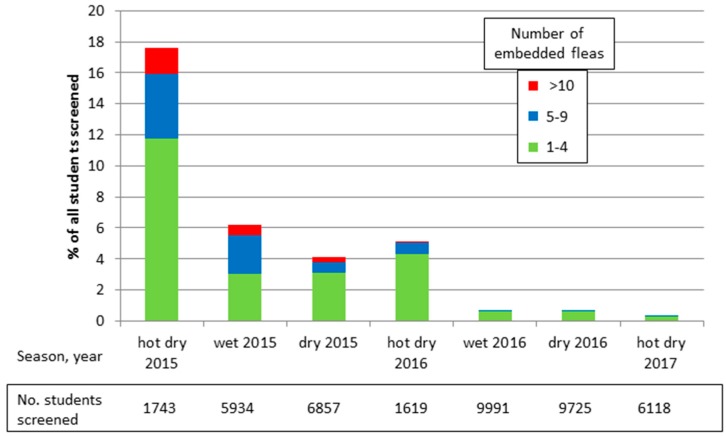
Period prevalence and intensity of tungiasis in target schools.

Figure 4 illustrates the foot screening records and how this simple community program has almost eliminated tungiasis from the schools targeted by the project. Initially 17.6% of all children screened in the target schools had at least one embedded sand flea, but after two years this was reduced to less than 1%. Severe tungiasis (>10 embedded fleas) seems to have been eliminated entirely, reducing from 2% in early 2015 to 0% in mid-2016, and moderate disease (5–9 embedded fleas) from 4% to 0.1%.

## 11. Discussion

Experience in endemic areas in South America and sub-Saharan Africa has shown that control of tungiasis, defined as elimination of severe, debilitating disease, is achievable through fairly simple and affordable community-based strategies incorporating both treatment and prevention components. Mapping of tungiasis is essential to understand exactly where transmission is occurring, in order to direct suitable interventions and to support advocacy efforts for funding and control programs.

The Tungiasis Eradication Project web mapping application was designed to be a workspace for aid workers, aid organizations, and governments of afflicted regions, channelling data from the local to the regional. The eLibrary project is an additional attempt to support global recognition of the disease. These web-based applications show that web GIS and VGI can readily assist health care providers in developing a cohesive control strategy for tungiasis worldwide. Multiple organizations can use these tools as collaborative workspaces with no barriers to sharing data. Such applications, backed by well-designed databases, can ensure standardized data collection across multiple groups separated by distance, budget, or language. 

A mixture of two dimethicones with low viscosity is currently the best option for treatment of human tungiasis, being highly effective with a single application. Dimethicone is currently only available in a few endemic areas. It is likely to also be effective for animal tungiasis, but given the larger volumes that would be required, treatment with a classical insecticide may be more economical [32].

Neem-based interventions deserve further investigation, since neem is widely available across the tropics and has been used for thousands of years to control insects [65]. Neem oil has been reported to kill arthropod skin parasites such as head lice (*Pediculus humanus capitis*, [66]), scabies *(Sarcoptes*) of both dogs [67] and sheep [68,69], and cattle ticks (*Boophilus microplus*) [70]. Studies have demonstrated neem extracts to be a powerful insect growth regulator, a feeding deterrent and repellent with low toxicity. It has been shown to control hundreds of arthropod species including agricultural pests and is used widely in organic farming as a natural pesticide [65]. The community in Kenya have found neem to be effective for human tungiasis and control of off-host stages, but it is also likely to be appropriate for use on animals. Since neem acts as a growth regulator, if it does not immediately kill the on- and off-host stages, it is likely to block the development and prevent egg-laying by the embedded flea, and thus block transmission. 

Animals can be treated with standard flea and tick control products, but most products are potentially toxic. They are relatively expensive and unlikely to be affordable for most affected families. In addition, veterinary services tend to be weak in most endemic countries and thus control using these products is unlikely. The community-based project in Kenya did not include a veterinary component, suggesting that at least in some areas, treatment of animals will not be necessary. It should be noted that in this particular area no animal reservoir has been identified yet, and prevalence among school children was less than 20% at the outset of the project. It is possible that in some areas transmission is purely anthropogenic and prevalence not so high. Where an animal reservoir has been shown to be involved, prevalence and intensity rates among the people correlate with that of the animals [30].

The use of simple insect repellents such as coconut oil may prevent infection and could be used to control disease, but have the disadvantage of needing to be applied twice a day, and may not be available in all endemic areas.

Currently there really is no effective and safe method to control off-host stages. All chemicals currently being recommended and used are highly toxic to humans, animals and the environment and are unaffordable for affected families. The only guaranteed safe and permanent method is to seal the house floors, but this is also currently unaffordable. New flooring technologies, such as that of Earthenable, need to be developed, made affordable, or subsidized to enable their adoption.

Although not yet evaluated as an intervention, any control campaign should include an element of community education. Most endemic communities can self-diagnose and are aware that the embedded fleas come from the floors of their home and surrounding area [37]. For many, all they need is to be taught how to protect themselves [42], and assisted to do so when necessary.

Since tungiasis affects the most marginalized families who rarely seek medical care, it needs to be addressed through a sustained community-based program led by public health departments. As demonstrated by the Kenyan example, a network of CHVs can conduct treatment and prevention education in schools and communities to effectively control disease. It is also quite feasible for the neem-based treatment and dimethicone to be distributed by CHVs, for family self-treatment. In fact, self-treatment would be preferable, since it would enable families to immediately treat a newly embedded flea before its rapid growth causes pain and before it starts to lay eggs.

However, it is unlikely that these strategies will lead to complete elimination of tungiasis. First of all, where reservoir hosts are involved, any environment can be re-contaminated by untreated reservoir animals, particularly where a sylvatic animal is involved. Likewise, the movement of infected people from areas without control efforts will re-establish infection, unless nationwide control is implemented.

Secondly, there are sub-groups within any population who are extremely hard to reach with control efforts. The most obvious are those who are marginalized by distance, but less obvious and more difficult to address, are those within a community who may never change their behaviour to protect themselves. These individuals include single parents (some of whom may be elderly grandparents caring for orphans), those who lack any education, those with mental or physical disability or alcohol and drug addiction. Some of these individuals are unable to absorb, process and act on information, while others are capable of understanding it, but incapable of acting on it. These are also the same individuals, or their children, found to have the most severe disease in any endemic population, before any control efforts start. 

For these cases there will need to be a more intensive effort by CHVs and public health officers (PHOs) to visit regularly to treat them and encourage them to adopt healthy practices. For those who cannot care for themselves, a family member or neighbour should be involved. For obstinate cases PHOs can recruit opinion leaders such as chiefs, elders and religious leaders to encourage behaviour change. Ultimately, the behaviour of a few individuals can perpetuate transmission to others and hinder control efforts.

With tens of millions of people estimated to be suffering with tungiasis in the Americas and sub-Saharan Africa, but with control clearly possible, there is an urgent need for research to evaluate and develop the various promising interventions. These include the neem-based treatments, scale-up of dimethicone use and the development of affordable hard floors. Global mapping of disease burden, vulnerable populations and transmission sites, and understanding the role of animal reservoirs in each area, will be essential to identify the target areas and suitable interventions.

## Figures and Tables

**Table 1:** URLs of the Tungiasis Elimination Project and eLibrary.

Tungiasis Elimination Project Web Page
http://www-scf.usc.edu/~kawright/Chigoe/ChigoeEradication.html
Tungiasis Elimination Project Map URL
http://uscssi.maps.arcgis.com/apps/webappviewer/index.html?id=4563a0de487046eb8c74eccfedc94ba0
Tungiasis eLibrary Web Page
http://www-scf.usc.edu/~kawright/Chigoe/TungiasiseLibrary.html
Tungiasis eLibrary Map URL
http://uscssi.maps.arcgis.com/apps/webappviewer/index.html?id=3f0a38ccd8c44ba7a6c4d58c4f24f6c8
